# Lab-scale tubular LED UV reactor for continuous photocatalysis

**DOI:** 10.1016/j.ohx.2023.e00506

**Published:** 2023-12-20

**Authors:** Stefan Herrmann, Lukas T. Hirschwald, Karl H. Heidmann, John Linkhorst, Matthias Wessling

**Affiliations:** a RWTH Aachen University, AVT.CVT – Chair of Chemical Process Engineering, Forckenbeckstr. 51, 52074 Aachen, Germany; b DWI – Leibniz Institute for Interactive Materials, Forckenbeckstr. 50, 52074 Aachen, Germany

**Keywords:** UV reactor, UV-LEDs, Photocatalysis, Tubular reactor, Water treatment

## Abstract

Photocatalytic water treatment is considered a promising technique to prevent micropollutants from entering the environment. However, no off-the-shelf UV reactors on lab scale are available to study new processes and photocatalysts. In this study, we present a tubular UV reactor equipped with 30 UV-LEDs, emitting UV light at 367 nm and a total radiant flux of 42 W. The UV reactor has an irradiated length of 300 mm and can host any transparent chemical reactor on the inside with a maximum diameter of 28 mm. The device is optimized for lab experiments with total dimensions of just 334 mm x 193 mm x 172 mm. Besides water treatment, a broad range of other photochemical and photocatalytic experiments can be performed with the reactor. Two identical UV reactors have been built and are successfully used for photocatalytic water treatment experiments. The degradation of methylene blue with TiO_2_ as photocatalyst was studied to validate the UV reactor. Furthermore, photocatalytic and hybrid processes were conducted in the UV reactor to degrade a broad range of pharmaceutical micropollutants.

## Graphical abstract

**Figure:**
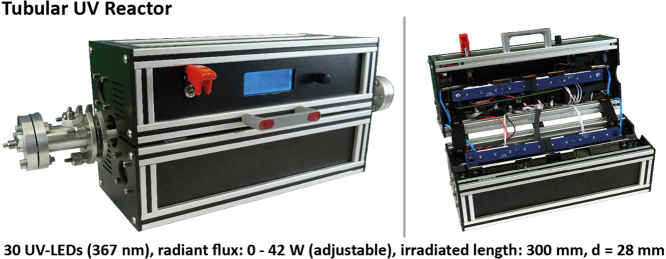

**Table:** 

**Specifications table**	

**Hardware name**	Tubular UV Reactor

**Subject area**	•*Chemistry and biochemistry* •*Environmental, planetary and agricultural sciences* •*General*

**Hardware type**	•*Chemical reactor, photo-reactor*

**Open source license**	Creative Commons Attribution 4.0 International License

**Cost of hardware**	1,246.60 € (Without 3D-printed parts, these can be ordered from an online manufacturer for around 85 €.)

**Source file repository**	https://doi.org/10.17605/OSF.IO/TRWZE

## 1 Hardware in context

Emerging global water scarcity and pollution threaten the fulfillment of the human right to safe and clean drinking water. The United Nations (UN) established 17 sustainable development goals, with goal 6 being clean water and sanitation [1]. However, not only in countries with severe problems in the drinking water supply but also in high-developed countries, water is nowadays contaminated with so-called micropollutants (MPs) [2], [3], [4]. These micropollutants are substances of anthropogenic origins, like industrial chemicals, pharmaceuticals, and personal care products, that appear at low concentrations. As an example, the widely used analgesic diclofenac can be found in a concentration range from ng L^-1^ to μg L^-1^ in the effluent of wastewater treatment plants [5], [6], [7]. Through the water cycle diclofenac can also make its way into groundwater, e.g., it was found on ng L^-1^ scale in different groundwater samples in Berlin (Germany) [8]. Classical wastewater treatment plants cannot remove MPs from water due to their persistent nature towards biological degradation. Hence, new treatment methods to encounter rising levels of MPs are necessary. One particularly promising strategy to degrade MPs is photocatalysis [9], [10], [11].

Many studies on photocatalytic water treatment use mercury-vapor lamps as a UV source in planar UV reactors [12], [13]. However, these lamps create a lot of waste heat, which is also fed into the process, changing the water temperature. On lab-scale, additional cooling for the water is necessary for experiments to prevent influences on MP degradation kinetics.

The ideal UV reactor for use in research needs to be able to host different photocatalytic processes. The waste heat by the UV lamps should be minimized, and the intensity of UV irradiation needs to be adjustable to screen process parameters. To increase the surface area to volume ratio and for a possible scale-up after lab-scale experiments, a tubular reactor should be favored. E.g., Jing et al. [14] and Ren et al. [15] showed that a tubular photo reactor concept is suitable for scale-up of photocatalytic hydrogen production. Besides classical challenges in reactor design, like residence time, flow properties, and mass transfer limitations, a new challenge occurs for photocatalytic reactors: the UV irradiation needs to be applied homogeneously to the tubular structure [16]. Thus, the design of the UV reactor becomes more important compared to the geometry of the water-carrying reactor parts [17].

UV-LEDs are a more energy-efficient source of UV radiation to build photocatalytic UV reactors. They have higher efficiencies than mercury-vapor lamps, thus reducing the energy input in the form of waste heat into the water stream [18], [19], [20]. Furthermore, reactors with UV-LEDs can be built smaller than one with mercury-vapor lamps, making them suitable for lab experiments [18]. However, most UV reactors using UV-LEDs are still of planar structure [21], [22], [23], [24], and just a few simple tubular or annular examples have been developed [20]. Furthermore, by replacing mercury-vapor lamps with LEDs, the UV source arrangement in the UV reactor becomes even more essential to ensure a homogeneous light distribution [20], [25].

This work presents a novel tubular UV reactor that uses UV-LEDs at a wavelength of 367 nm for the degradation of MPs on lab-scale. The LEDs are arranged in six planar array rows of 5 LEDs each. Two arrays are placed in a row and three of these rows are distributed on the circumference of the reactor tube spaced by 120° to irradiate a tubular flow reactor from all sides. We use a tubular Al_2_O_3_ membrane coated with TiO_2_ as photocatalyst for the degradation of MPs. The membrane is placed in a fused silica housing with a high UV transmittance.

The advantage of the UV reactor presented in this work, compared to other UV reactors, is its flexibility. It can be used for any tubular geometry and reactor configuration that fits into the measures of the reactor. Thus, three-phase reactions with additional gas phases, homogeneous as well as heterogeneous photocatalysis, are possible with this UV reactor. It could even serve as a continuous UV cross-linking reactor for (membrane) fibers after the spinning process.

## 2 Hardware description

This work presents a tubular UV reactor for a broad range of applications. Even though designed for photocatalytic water treatment, it can be used for other photochemical and photocatalytic reactions. The UV reactor is equipped with 30 UV-LEDs with a wavelength of 367 nm. The total radiant flux of the LEDs is 42 W with an electrical power consumption of 111 W. The radiant flux can be adjusted by pulse width modulation (PWM) to reduce the irradiation intensity. The reactor can host any tubular reactor geometry with a maximum diameter of 28 mm and provides an irradiated length of 300 mm, giving it great flexibility for lab-scale research.

**Fig. 1:**
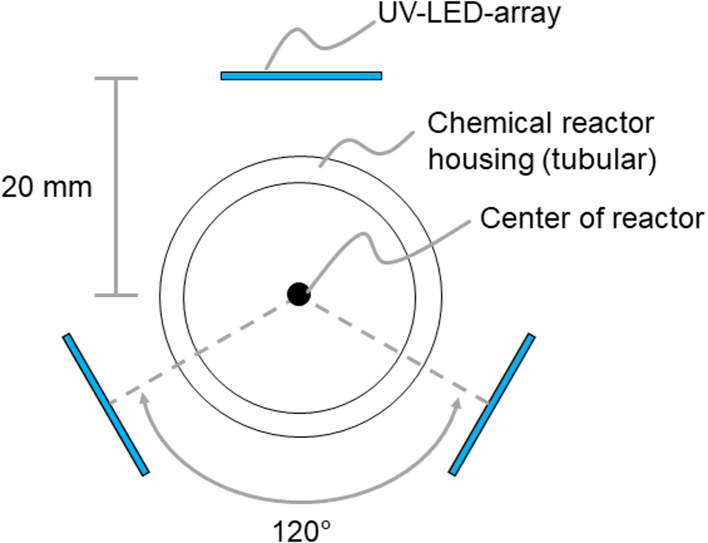
Illustration of the relative positions of the UV-LED-arrays in the UV reactor. The distance of the arrays to the center is 20 mm and the three arrays are arranged with an offset angle of 120°.

The UV-LEDs are arranged in linear arrays of five LEDs. The distance between the LEDs is designed to completely irradiate a cylindrical target surface with a diameter of 10 mm in the center of the UV reactor, respecting the LED’s radiation angle of 115°. Two of these UV-LED-arrays are arranged linearly within the reactor to provide a total length of 300 mm. Three of these UV-LED-arrays are arranged around the circumference with an offset angle of 120° to irradiate the target surface homogeneously, as shown in Fig. 1.

**Fig. 2:**
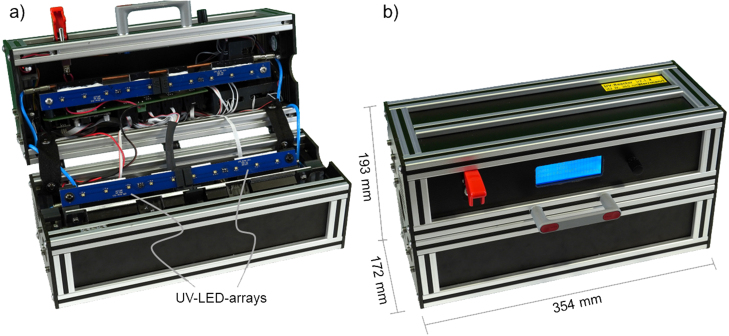
Pictures of the UV reactor in the configuration with additional water cooling. (a) Open. (b) Closed.

The reactor uses minimal lab area with a footprint of just 354 mm x 193 mm x 172 mm, including all electronics except for the power supply. As a safety feature, a hall sensor and a magnet are used to indicate an opened reactor. If the software detects the reactor is opened, the LEDs are immediately turned off to protect the user from UV radiation. Fig. 2 shows the open and closed UV reactor. All parts are either off-the-shelf or custom-made but easy to order online with the respective design files, like printed circuit boards (PCBs) for electronics. All files necessary to rebuild the reactor are available with this work. Overall costs to build the UV reactor were below 1,250 € without the expenses for 3D-printed parts as a 3D printer was available and material costs were negligible.

Our tubular reactor design offers several benefits over state-of-the-art solutions:

• A cheap, lab-scale reactor for photocatalytic advanced oxidation processes for water treatment.
• Easy screening of process parameters like irradiation intensity.
• A reactor suitable for homogeneous or heterogeneous photocatalysis in tubular chemical flow reactors.
• The reactor is suitable for other applications, such as cross-linking continuous fiber production.

## 3 Design files summary

All files to build the presented UV reactor are available from the open science framework repository (see Table 1). The housing is made from a standard aluminum system profile (ITEM Industrietechnik GmbH, Germany) closed with plastic panels. Mountings for the UV-LED-arrays, main PCB, display, constant current power supplies, and the tubular reactor are 3D-printed. All 3D-printed files are available as .stl and .stp files in the repository. Technical drawings for the assembly of the system profiles, processing of the plastic panels, and processing of the heat sinks are also uploaded. The design of the UV-LED-array PCBs and the main PCB are available as KiCad files and Gerber files. The circuit diagrams are available as .pdf files and the Arduino software to operate the UV reactor is published in the repository.

**Table 1:** Design files for rebuilding the UV reactor.

Design filename	File type	Open source license	Location of the file
3D_printed_mountings			

Display_mounting	CAD files (.stl, .stp)	CC-BY 4.0	OSF Repository
Hall-sensor_Mounting	CAD files (.stl, .stp)	CC-BY 4.0	OSF Repository
LED-array_mounting_bottom_middle	CAD files (.stl, .stp)	CC-BY 4.0	OSF Repository
LED-array_mounting_bottom_side	CAD files (.stl, .stp)	CC-BY 4.0	OSF Repository
LED-array_mounting_top_middle	CAD files (.stl, .stp)	CC-BY 4.0	OSF Repository
LED-array_mounting_top_side	CAD files (.stl, .stp)	CC-BY 4.0	OSF Repository
Mounting_glass_tube	CAD files (.stl, .stp)	CC-BY 4.0	OSF Repository
PCB_main_distance	CAD files (.stl, .stp)	CC-BY 4.0	OSF Repository
Power_supply_distance	CAD files (.stl, .stp)	CC-BY 4.0	OSF Repository
Power_supply_housing_2	CAD files (.stl, .stp)	CC-BY 4.0	OSF Repository
Power_supply_housing_4	CAD files (.stl, .stp)	CC-BY 4.0	OSF Repository

Technical_drawings			

Heat_sink_large_drawing	CAD files (.dwg, PDF)	CC-BY 4.0	OSF Repository
ITEM_panels	CAD files (.dwg, PDF)	CC-BY 4.0	OSF Repository
ITEM_UV_reactor_bottom	CAD files (.dwg, PDF)	CC-BY 4.0	OSF Repository
ITEM_UV_reactor_top	CAD files (.dwg, PDF)	CC-BY 4.0	OSF Repository

PCBs			

Circuit_diagram	PDF file	CC-BY 4.0	OSF Repository
PCB_main	PDF file	CC-BY 4.0	OSF Repository
PCB_main	KiCad, Gerber	CC-BY 4.0	OSF Repository
PCB_UV-LEDs	PDF file	CC-BY 4.0	OSF Repository
PCB_UV-LEDs	KiCad, Gerber	CC-BY 4.0	OSF Repository

Arduino_software			

Software_3.1	Arduino (.ino)	CC-BY 4.0	OSF Repository

**Table 2:** Bill of materials for the UV reactor.

Designator	Component	#	Cost per	Total	Source of materials
			unit in €	cost in €	
Housing	Details s. Table 3	1		a433.60	tinyurl.com/yn2ekr68
PCBs	PCB_main	1	6.50	6.50	tinyurl.com/4dfmbu5u
	Al-PCB_UV-LEDs	6	13.30	79.80	tinyurl.com/3t3393e8
	(3 W/mK)				
	Stencil: UV-LEDs	1	59.00	59.00	tinyurl.com/3t3393e8
UV-LEDs	Seoul Viosys	30	7.50	225.00	tinyurl.com/2jp6rjh9
	CUN6GF1A				
Power supply (CC)	Lumitronix	6	14.90	89.40	tinyurl.com/267kery7
	1000 mA				
Heat sink small	Fischer	6	1.50	9.00	tinyurl.com/yfz9593x
	ICK BGA 23 × 23				
Heat sink large^b^	Fischer	2	9.00	18.00	tinyurl.com/4xnutwj4
	SK 81/ 75 SA				
Adhesive	Heat conductive	1	30.20	30.20	tinyurl.com/2s3srejs
	TBS20S				
Arduino	Nano v3	1	23.50	23.50	tinyurl.com/4ax9uy7c
Display	DEBO LCD 20X4 BL	1	14.70	14.70	tinyurl.com/4raay75n
Stepdown 12V	LM2596S DC-DC	1	2.00	2.00	tinyurl.com/556mphpx
Hall sensor	DEBO SENS HALL	1	4.50	4.50	tinyurl.com/3963rhu2
Magnet	d = 5 mm	1	0.20	0.20	tinyurl.com/y4c9mted
Rotary encoder	DEBO ROT SWITCH	1	2.70	2.70	tinyurl.com/yu4jtstx
Screw terminal	TE AMP 282837–2	1	1.00	1.00	tinyurl.com/5cyj3n6u
IDC connector	T816 male, 6 pin	19	0.50	9.50	tinyurl.com/ys7px655
	T816 female, 6 pin	19	0.80	15.20	tinyurl.com/ztefb7p3
Fan 40 × 40 mm	Sunon	4	3.00	12.00	tinyurl.com/4zjvhr9c
	FAN-ML 4010–12				
Ribbon cable	3M 3801–10	10 m	1.60	16.00	tinyurl.com/55nes89j
Power switch	KS C3900	1	8.30	8.30	tinyurl.com/bdh2brmx
Switch cover	GUARD R17–10	1	3.70	3.70	tinyurl.com/54rmtj5z
Power supply 24 V	MW GST220A24	1	87.50	87.50	tinyurl.com/2p964wpk
Power socket	DIN 4 pin R7B, female	1	2.00	2.00	tinyurl.com/4vvb9krr
Nuts	DIN 934 M5 × 6	4	0.10	0.40	tinyurl.com/29uk6rdc
	DIN 934 M4 × 6	16	0.10	1.60	tinyurl.com/mr2swfkh
Washers	DIN 125 M3	1	0.50	0.50	tinyurl.com/yzbb5fu6

a Bulk price for whole assembly incl. cutting.

b Additional processing/cutting required.

**Table 3:** Bill of materials for the UV reactor housing from item24.de.

Designator	Component	Article No.	#	Length	Material type
		(item24.de)		mm	
Top part					

System profiles	Profile 5, 20 × 20 mm	0.0.370.03	3	51	Aluminum
	Profile 5, 20 × 20 mm	0.0.370.03	4	56	Aluminum
	Profile 5, 20 × 20 mm	0.0.370.03	2	100	Aluminum
	Profile 5, 20 × 20 mm	0.0.370.03	1	310	Aluminum
	Profile 5, 20 × 20 mm	0.0.370.03	4	350	Aluminum
Handle	Pi 80 M5 PA, grey	0.0.679.07	1	–	Plastic
Standard-fastening set	Profile 5	0.0.370.08	17	–	Zinc-plated

Bottom part					

System profiles	Profile 5, 20 × 20 mm	0.0.370.03	3	50	Aluminum
	Profile 5, 20 × 20 mm	0.0.370.03	4	56	Aluminum
	Profile 5, 20 × 20 mm	0.0.370.03	2	58	Aluminum
	Profile 5, 20 × 20 mm	0.0.370.03	2	100	Aluminum
	Profile 5, 20 × 20 mm	0.0.370.03	1	310	Aluminum
	Profile 5, 20 × 20 mm	0.0.370.03	4	350	Aluminum
Standard-fastening set	Profile 5	0.0.370.08	19	–	Zinc-plated

Panels					

Panel 4 mm	320 × 66 mm^a^	0.0.474.37	4	–	Plastic
black/RAL9017	320 × 50 mm^a^	0.0.474.37	4	–	Plastic
	140 × 96 mm^a^	0.0.474.37	4	–	Plastic

General					

Hinge	5 PA, black	0.0.370.18	3	–	Plastic
Cover profile	5, black	0.0.391.74	6	2000	Plastic
T-slot nuts	5 St M5	0.0.370.01	8	–	Zinc-plated
T-slot nuts	5 St M3	0.0.437.19	2	–	Zinc-plated
Screws	DIN 912 M5 × 18	0.0.655.05	1	–	Zinc-plated
	DIN 912 M5 × 12	8.0.004.59	22	–	Zinc-plated
	DIN 912 M5 × 8	0.0.609.72	5	–	Zinc-plated
	DIN 912 M4 × 20	8.0.000.23	16	–	Zinc-plated
	DIN 912 M3 × 20	0.0.616.29	2	–	Zinc-plated

a Additional processing/cutting required.

**Table 4:** Bill of materials for the optional water cooling system.

Designator	Component	#	Cost per	Total	Source of materials
			unit in €	cost in €	
Push-in connector	Sang-A IQSS 60 MSV	2	5.40	10.80	tinyurl.com/ys79pz9m
Push-in manifold	Sang-A IQSQ 6040	2	5.70	11.40	tinyurl.com/25c5hmyp
Push-in fitting	Sang-A IQSG 40 MSV	6	3.10	18.60	tinyurl.com/32brydyb
Push-in valve	Sang-A IQSKH 60	2	8.60	17.20	tinyurl.com/yfh4v3b3
Copper tubing	4 × 1 mm	2 m	13.35	26.70	tinyurl.com/ua8rcxz6
PU tubing	Sang-A PUN 4X2,5	2 m	0.70	1.40	tinyurl.com/2s4hkw92
PU tubing	Sang-A PUN 6X4	1 m	0.70	0.70	tinyurl.com/26xppuev
Washers	DIN 125 M4	2	2.00	4.00	tinyurl.com/4tayxk54

**Table 5:** Bill of materials for 3D-printed parts for the UV reactor. The heat-resistant parts were produced by stereolithography (SLA) on a Form 3 3D printer with HighTemp resin (Formlabs Inc., USA). All other parts were fabricated via fused filament fabrication (FFF) from polyethylene terephthalate modified with glycol (PETG) on a Prusa i3 MK3S+ (Prusa Research a.s., Czech Republic). When ordering the parts from an online manufacturer, all parts can be printed via FFF from acrylonitrile butadiene styrene (ABS). The cost is around 85 €.

Designator	File name	#	Material type
LED-array mounting bottom	LED-array_mounting_bottom_middle.stl	1	Plastic (heat-resistant 80 °C)
	LED-array_mounting_bottom_side.stl	2	Plastic (heat-resistant 80 °C)
LED-array mounting top	LED-Array_mounting_top_middle.stl	1	Plastic (heat-resistant 80 °C)
	LED-array_mounting_top_side.stl	2	Plastic (heat-resistant 80 °C)
PCB_main distance	PCB_main_distance.stl	2	Plastic
CC Power supply mounting	Power_supply_housing_4.stl	1	Plastic (heat-resistant 80 °C)
	Power_supply_housing_2.stl	1	Plastic (heat-resistant 80 °C)
	Power_supply_distance.stl	1	Plastic
Display mounting	Display_mounting.stl	1	Plastic
Hall sensor mounting	Hall sensor_mounting.stl	1	Plastic
Tubular reactor mounting	Mounting_glass_tube.stl	2	Plastic

## 4 Bill of materials summary

All components to build the UV reactor are listed in Table 2, Table 3, Table 4, Table 5. Table 2 lists the main components of the UV reactor that are commercially available. A detailed description of the system profiles for the reactor housing and the plastic panels is shown in Table 3. Table 4 lists all parts necessary for the additional water cooling option of the reactor. Table 5 lists all custom-made parts that are 3D-printed. Some parts, such as the plastic panels and the large heat sinks, require additional manufacturing steps with a milling machine, laser cutter, or saws after purchase. Furthermore, a reflow oven is needed to solder the UV-LEDs onto the corresponding PCBs. A M5 thread cutter is necessary to assemble the system profiles and plastic panels. All 3D-printed parts were painted after printing for visual reasons.

## 5 Build instructions

Three main parts are required to build the UV reactor: the housing, the UV-LED-arrays, and the control and power electronics of the reactor. Finally, all main parts are assembled. The construction is explained according to this structure.

### Housing.

The housing is made from aluminum system profiles of ITEM Industrietechnik GmbH, Germany. All components for the top and bottom parts of the housing can be ordered with ITEM to the above specifications, i.e., correct length and drilled holes for the standard fastening sets used to connect the profiles. The profiles can be purchased from any other manufacturer of similar aluminum profiles. The amount and length of the profiles for the housing are listed in Table 3.

The plastic panels were also ordered with ITEM, but every other plastic panel supplier is also suitable. The ordered measures of the panels are listed in Table 3. The panels need post-processing with a milling machine, laser cutter, or saw. The final dimensions for all panels are shown in the technical drawing ITEM_panels.pdf. All edges must be cut out to allow space for the fastening sets to connect the profiles. If other profiles and fastening methods are used, the measures for the cutouts may differ. Also, cutouts for the power switch, rotary encoder, display, fans, water cooling, power supply, and the tubular reactor module are necessary.

**Fig. 3:**
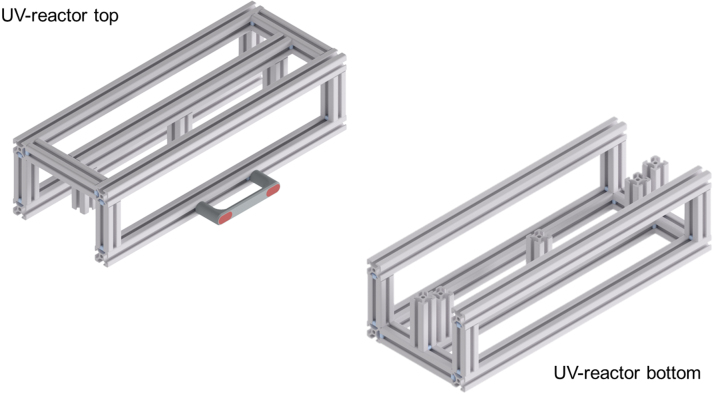
Rendering of the top and bottom half of the assembled aluminum system profile.

**Fig. 4:**
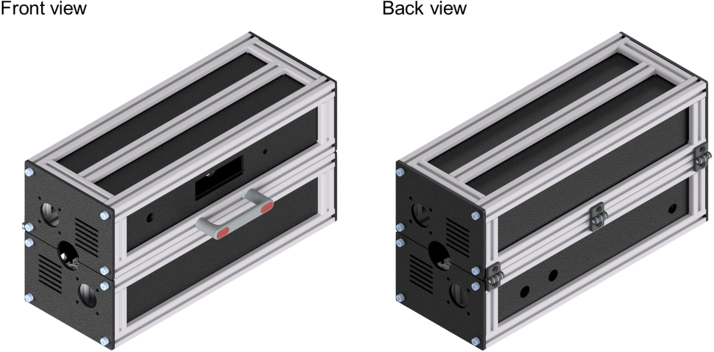
Rendering of the assembled housing with panels, handle, and hinges.

The assembly of the profiles is done, as shown in Fig. 3, according to the technical drawings ITEM_UV_Reactor_bottom.pdf and ITEM_UV_Reactor_top.pdf. During the assembly, the plastic panels are inserted in the profile slots. ITEM cover profiles are inserted into the slots to ensure secure mounting before adding the panels. The panels covering the sides are connected to the housing with M5 × 12 screws. An M5 thread must be cut into the center holes of the corresponding profiles beforehand. Both halves of the reactor housing are connected with three hinges on the backside. A handle is mounted to the front side of the top half to open and close the housing, as shown in Fig. 4. All slots on the outside of the housing are covered with black ITEM cover profiles to improve the visual appearance of the UV reactor and prevent dirt from accumulating.

**Fig. 5:**
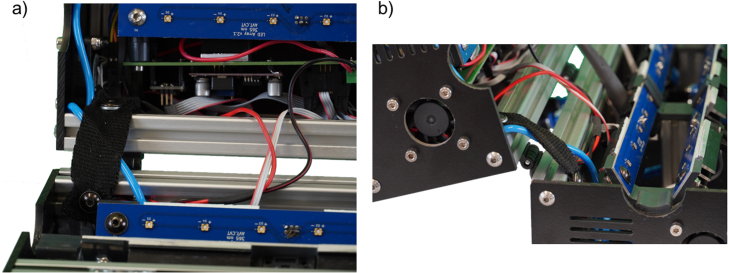
Pictures of the strap, preventing an opening angle of more than 100°. (a) Front view. (b) Side view.

As shown in Fig. 5 two straps are mounted on the back inside the reactor between the top and bottom halve to prevent the top from opening more than 100°. The straps are mounted with M5 screws and T-slot nuts. Furthermore, a heavy metal block of approximately 1 kg is placed in the front of the UV reactor’s inside to prevent the device from tilting when the top part is opened. As these are optional features and were realized with leftover materials from other projects and the mechanical workshop, these parts are not listed in the BOM.

### UV-LED-arrays.

Five UV-LEDs are soldered onto an aluminum PCB to form a UV-LED-array. A UV reactor consists of six of these arrays. The PCB was designed in KiCad and ordered as aluminum PCBs with increased heat conductivity of 3 W/mK and a thickness of 1.6 mm from LeitOn, Germany. The UV-LEDs were soldered onto the PCB in a reflow oven. A stencil was directly ordered with the PCBs and used to apply the soldering paste. The reflow soldering process was done according to the technical data sheet of the LEDs. The UV-LED-arrays can also be ordered with assembled LEDs if a reflow oven is not available.

A male 6-pin IDC T816 connector is soldered to the back side of the PCB to allow connection to the control and power electronics with a ribbon cable. These connectors are used for all electronic connections in the UV reactor to reduce the number of different parts.

**Fig. 6:**
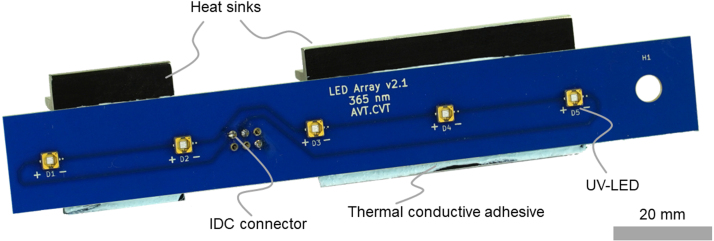
Assembled UV-LED-array with five UV-LEDs, two heat sinks, and a male IDC T816 connector.

Two different heat sinks are placed next to the IDC T816 connector on the back of the PCB to dissipate the waste heat of the UV-LEDs. A 25 mm and a 57 mm long heat sink with a width of 29 mm and five fins is needed for each UV-LED-array. These are manufactured with a milling machine or a saw from the large heat sink (Fischer SK 81/75 SA) as raw material, according to technical drawing Heat_sink_large_drawing.pdf. After manufacturing the heat sinks, they are mounted onto the back of the PCB with the heat conductive adhesive from Table 2. Fig. 6 shows the final UV-LED-array with two heat sinks and an IDC T816 connector.

### Control and power electronics.

**Fig. 7:**
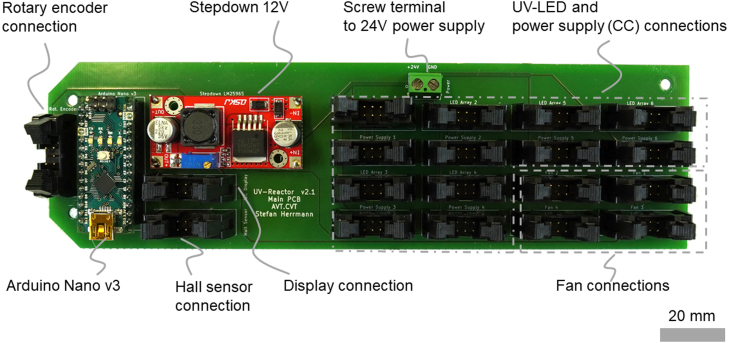
Picture of the main PCB with the Arduino Nano v3, 12 V stepdown, screw terminal, and 19 IDC T816 connectors to connect the rotary encoder, hall sensor, display, fans, CC power supplies, and the UV-LED-arrays.

Each UV-LED-array needs a current of 1 A and a voltage of 18.5 V as five LEDs are connected in series. Hence, each UV-LED-array is connected to a corresponding power supply to provide the current. Standard 1000 mA constant current (CC) power supplies for LEDs are used. These CC power supplies can also change the irradiation intensity by PWM. All six CC power supplies are connected to the main PCB with ribbon cables and IDC connectors. Five of the six pins are used for input voltage, PWM signal, and output current. The main PCB is connected to a 220 W, 24 V power supply that is placed outside of the UV reactor. Thus, creating a 24 V grid on the main PCB. Fig. 7 shows a picture of the main PCB. A power switch with a switch cover is placed between the external power supply and the main PCB. A 12 V stepdown module is used to provide an additional 12 V grid for all control electronics on the main PCB. Four fans are used to cool the UV reactor on the inside. The fans are connected to the main PCB’s 12 V grid for power supply and start running when the device is switched on. All pin assignments are described in detail in the circuit diagrams that are uploaded to the repository.

An Arduino nano v3 is used to control the UV reactor. It is placed on the main PCB. A four-line display is used to visualize the control menu and the current status of the UV reactor. A rotary encoder with a push button is used to navigate the menu. Within the menu, the irradiation intensity and time of irradiation can be selected before the UV-LEDs are switched on. The Arduino software to control the UV reactor is available in the repository. The software is uploaded to the Arduino before the final assembly of the UV reactor.

The UV reactor uses UV radiation of high intensity, which can cause severe damage to human skin. Thus, the reactor needs to immediately switch off the UV-LEDs when it is opened. As a safety feature, a hall sensor and a magnet detect any reactor opening. If the magnet is removed from the hall sensor, the Arduino will switch off all UV-LEDs.

All electronic components are connected to the main PCB with IDC T816 connectors, except for the main power supply that uses a screw terminal on the main PCB and a DIN 4-pin R7B connector at the UV reactor housing to connect to the external 24 V power supply. Overall, the main PCB consists of an Arduino, a 12 V stepdown module, 19 IDC T816 connectors (male), and a screw terminal. All parts are soldered onto the main PCB.

### Final assembly.

**Fig. 8:**
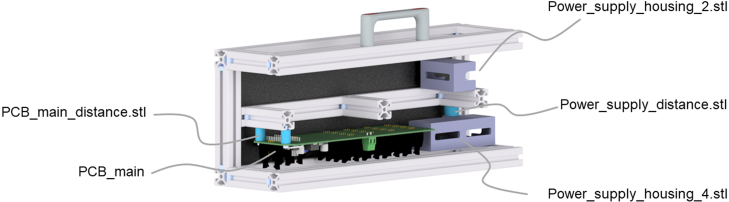
Rendering of the top half of the reactor to show the position of the main PCB and the power supply housings. Power supplies, cables, and all components on the main PCB are not shown for simplification reasons.

After the UV reactor’s housing assembly, the four fans are connected to the side panels with M4 × 20 screws and M4 × 6 nuts. The open cable ends of the fans are connected to female IDC T816 connectors. The assembled main PCB is connected to all 19 ribbon cables with IDC T816 connectors. The screw terminal is connected to the wires for the power supply. Then the PCB is placed into the left top half of the UV reactor, as shown in Fig. 8, and connected to the system profile with two M3 × 20 screws, two M3 T-slot nuts, and two PCB_main distance bushings to adjust the position in the reactor. The CC power supplies are combined with the related small heat sinks (Fischer ICK BGA 23 × 23) by using the heat conductive adhesive. Next, the CC power supplies are connected to the open ends of the corresponding ribbon cables by soldering and then placed into the power supply housings. One housing can host four CC power supplies, and the other can host the remaining. The power supplies are just pushed into the housings. The power supplies are installed in the top right half of the UV reactor, as shown in Fig. 8. The housing with two power supplies is directly screwed to the system profile with an M5 × 8 screw and an M5 T-slot nut. The housing with four power supplies is screwed to the system profile with an M5 × 18 screw, an M5 T-slot nut, and the power supply distance bushing.

**Fig. 9:**
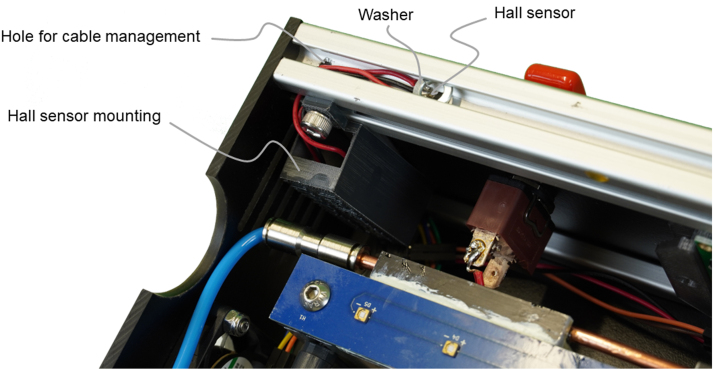
Picture of hall sensor and housing position. The existing hole to tighten the system profile was used for proper cable management.

The display is mounted to the front panel with the display mounting bracket and glue. The rotary encoder and the power switch with its cover are screwed into the front panel. All pins of these parts are connected to the corresponding ribbon cables by soldering. The hall sensor is desoldered from its PCB and extended with two wires. The PCB of the hall sensor is placed into the 3D-printed hall sensor housing. The housing is screwed to the system profile with M5 × 8 screws and M5 T-slot nuts. The hall sensor itself is placed into the front slot of the system profile, as shown in Fig. 9. The wires are clamped in the slot with a washer. A magnet is glued in the slot of the bottom half of the reactor, directly opposite the hall sensor.

The DIN 4-pin R7B socket is glued on the back panel of the reactor’s bottom half. Thus, the external power supply can easily be connected to the UV reactor. The socket is connected to the power switch and the screw terminal of the main PCB with wires of sufficient diameter for up to 10 A and 24 V.

**Fig. 10:**
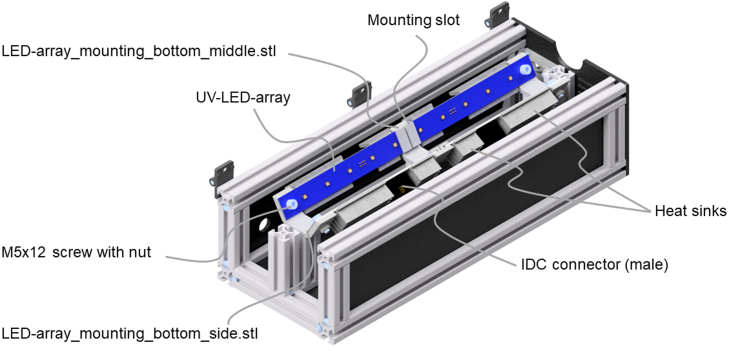
Rendering of the bottom half of the reactor to show the mounting of the LED-arrays with the 3D-printed mountings.

The 3D-printed LED-array mountings are glued onto the six system profiles in the middle of the reactor to install the UV-LED-arrays. After drying, the final assembled LED-arrays can be put into the slot of the middle mounting and screwed into the mountings on the sides of the reactor with M5 × 12 screws and M5 × 6 nuts, as shown in Fig. 10. The nuts are only necessary at the bottom half. At the top half, M5 threads need to be cut into the system profile’s center hole. The UV-LED-arrays are connected to their respective ribbon cables with IDC T816 connectors.

Finally, the 3D-printed tubular reactor mountings are glued onto the outer system profiles on the bottom half of the reactor. The geometry is optimized to host reactors with a diameter of 28 mm inside the UV reactor. If other reactor diameters are used, this part needs to be adjusted to position the reactor in the center of the circular-arranged UV-LED-arrays.

### Optional water cooling.

The reactor can be built and operated as explained above. However, the UV-LEDs may encounter elevated temperatures near their maximum operating temperature. To increase the lifetime of the LEDs, an additional water cooling system was implemented in the UV reactor after six months of smooth operation without a water cooling system.

**Fig. 11:**
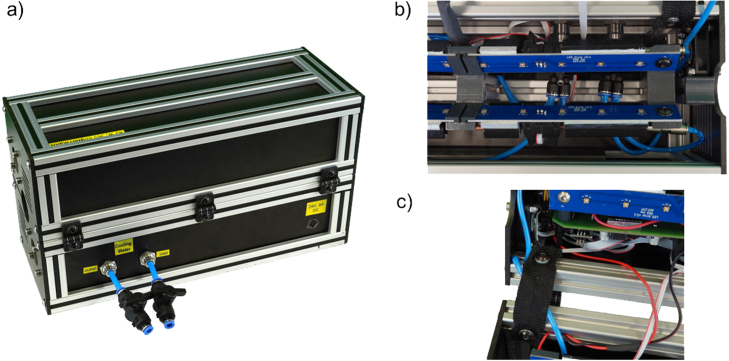
Pictures of the optional water cooling in the UV reactor. (a) Rear view of the reactor with inlet and outlet for cooling water, connected to respective valves. (b) Push-in manifolds of the water cooling system. (c) Detail of the tube mounting in the slot with a washer for the top part of the reactor.

To install the water cooling, two additional 15 mm holes are drilled into the bottom back panel of the UV reactor, as shown in technical drawing ITEM_panels.pdf and in Fig. 11 (a). Two panel mount push-in connectors (IQSS 60 MSV) are mounted in these holes. On the inside of the reactor, a short piece of 6 mm tubing is connected to the push-in connectors, and the push-in manifolds (IQSQ 6040) are connected to the other side of the tubes, as shown in Fig. 11 (b). One manifold operates as an inflow and the other as the return flow manifold. A straight copper tube with an outer diameter of 4 mm is placed between two fins of the heat sinks of each UV-LED-array and fixed with heat conductive adhesive. A 4 mm push-in fitting (IQSG 40 MSV) is placed on each end of the copper tubes. Each copper tube is connected with 4 mm tubing to the inflow manifold on one side and the return flow manifold on the other side. To allow smooth opening and closing of the reactor, the tubing for the top part of the reactor is mounted in a T-slot with the washers, as shown in Fig. 11 (c). The fourth openings of the manifolds are either shorted or closed, depending on the inlet pressure of the cooling water. It is recommended to shorten the openings for high inlet pressures to prevent leakages at the push-in fittings in the reactor. The panel mount push-in connectors outside the reactor are connected to valves (IQSKH 60) and the cooling water circuit. The valves should be closed before disconnecting the cooling water circuit. A proper leak test must be conducted before connecting the reactor to electricity.

After installing the water cooling system, tests with our in-house cooling water circuit (inflow at 4°C and 3.0 bar) were conducted. The hot-spot temperature of the UV-LED-arrays dropped by up to 20°C. Thus, the lifetime of the LEDs should be significantly extended.

## 6 Operation instructions

To use the UV reactor, first, the chemical reactor is placed inside on the two tubular reactor mountings. The chemical reactor should be placed in the center of the UV-LED-arrays and needs to be made from a material of high UV transmittance. In the case of this work, a fused silica tube with an outer diameter of 28 mm is used. The reactor is closed afterward and switched on with the power switch on the front panel. The Arduino starts up, and the display shows the setup page.

A drawback of the current start-up procedure is that the UV-LEDs are switched on for approximately 1 s when the UV reactor is switched on. The reason for this is the UV-LED control with the PWM signal of the Arduino. The signal is set to zero after the Arduino start-up procedure is finished. Thus the UV-LEDs are on for the time of the start-up. To overcome this drawback, the UV-LEDs can be controlled with additional relays. However, for simplification reasons, together with a risk assessment of the UV radiation at 367 nm this was not done in this work. It is highly recommended to change this when working with UV-LEDs of lower wavelength (e.g., 254 nm).

**Fig. 12:**
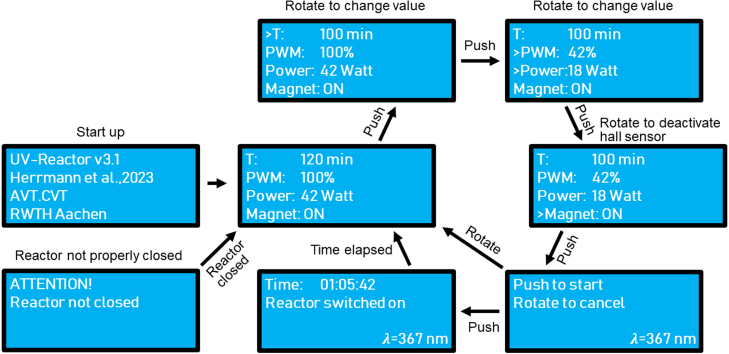
Flow diagram view of the UV reactor’s operation menu.

After the start-up of the UV reactor, the operating parameters need to be set. Fig. 12 shows the workflow of the operation menu. By pushing the rotary encoder, the selection of the irradiation time starts. The time can be changed by turning the rotary encoder. A further push selects the displayed value, and the menu switches to the selection of the irradiation intensity. The intensity can be adjusted between 0 to 100%. The display also shows the radiant flux between 0 to 42 W. The intensity can be changed by turning the encoder and is selected by pushing it. Lastly, the query of the hall sensor status can be turned off, e.g., for maintenance or inspections of the UV reactor. The UV reactor needs to be handled with caution when this mode is selected, as the UV-LEDs will not turn off when the reactor is opened. Appropriate personal protective equipment should be worn. The hall sensor status is confirmed by pushing the rotary encoder. For safety reasons, a second push is needed to start the irradiation. The display shows “push to start” and “rotate to cancel”. When the rotary encoder is pushed, the UV-LEDs are immediately switched on. The menu jumps back to the previous page if the rotary encoder is rotated. When the UV-LEDs are switched on, the display shows the remaining irradiation time and gives the information that the reactor is switched on.

If the reactor is opened during operation, the LEDs are switched off immediately, and the display reports an error that the UV reactor is not properly closed. It needs to be closed to switch the UV reactor back on, and the operating parameters must be set up again before starting. The UV reactor can be turned off with the power switch at any time without damaging the device. If increased safety measures are necessary, e.g., when UV-LEDs with a wavelength of 254 nm are used, a physical switch can be used instead of a hall sensor to monitor the proper closing of the reactor. That way, the UV-LEDs are switched off independent of the Arduino and its software when the UV reactor is opened.

If the UV reactor is equipped with the optional water cooling, the valves need to be opened, and the cooling water flow needs to be started before the UV-LEDs are switched on. It is recommended to keep the cooling water flowing for several minutes after shutting down the reactor at the end of the experiments.

## 7 Validation and characterization

**Fig. 13:**
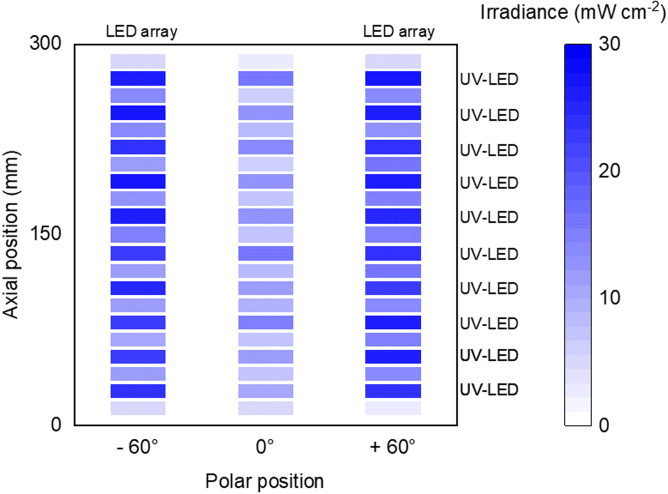
Measured UV irradiance in the center of the UV reactor (distance to the UV-LED-array: 20 mm). The measurement was done at the bottom half of the UV reactor at a radiant flux of 12.6 W.

The overall power consumption with the water cooling system is measured during operation to characterize the wall-plug efficiency of the UV reactor. At a radiant flux of 12.6 W and 42 W, the power consumption is 44.0 W and 131.2 W, respectively. This results in a wall-plug efficiency of 29.5% and 32.0%, respectively. Furthermore, the irradiance in the center of the UV reactor as a function of axial and polar position was measured with an IM-213 UV-AB meter (RS Components Ltd., United Kingdom). The irradiance was measured in the bottom half of the UV reactor at the polar positions of the UV-LED-arrays (−60 ° and +60 °) and in between both arrays (0 °). In axial direction, the irradiance was measured at the positions of the UV-LEDs and in the middle between two UV-LEDs. Due to the UV meter’s measuring range of 0 to 40 mW cm^-2^, all measurements were done at a radiant flux of 12.6 W. Fig. 13 shows the results of the irradiance measurement. As expected, the irradiance changes with the position in the UV reactor. However, the irradiance is above 10 mW cm^-2^ in a predominant area of the UV reactor. Additional reflectors could be added to the UV reactor to create a more homogeneous irradiance.

The UV reactor was designed for water treatment experiments. The degradation of methylene blue (MB) as a model component is studied to prove the UV reactor is suitable for photocatalytic degradation in water treatment. MB can be degraded by TiO_2_ but is not degraded by UV irradiation on its own [26]. A tubular reactor made from transparent poly methyl methacrylate (PMMA) with an outer diameter of 10 mm was equipped with 3 TiO_2_-PVDF fibers. The fibers contained 36-wt% TiO_2_ as photocatalyst. The PMMA reactor was placed inside the UV reactor with adjusted tubular reactor mountings to position the PMMA reactor in the center of the UV-LED-arrays.

**Fig. 14:**
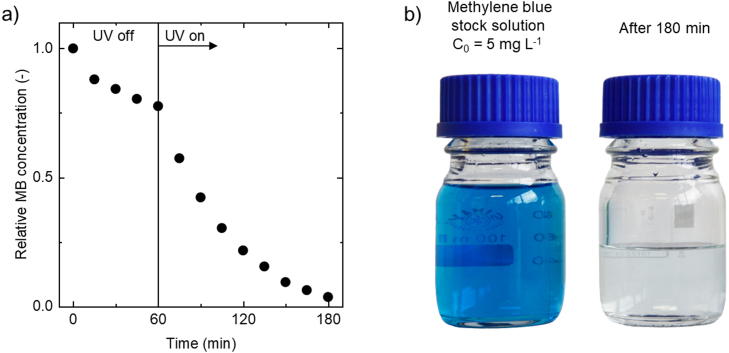
Methylene blue (MB) degradation with TiO_2_-PVDF fibers in the UV reactor at 30% irradiation intensity. The fibers contain 36-wt% TiO_2_ as photocatalyst. A MB solution (5 mg L^-1^ in water) is pumped through the tubular flow reactor. (a) Relative MB concentration over time, measured by UV–Vis spectroscopy. (b) MB stock solution and discolored solution at the end of the experiment.

A stock solution was prepared with 5 mg L^-1^ MB in ultra-pure water (MilliQ). Then, 100 mL of this stock solution were pumped through the module for 180 min in a closed-loop with a reservoir at a flow rate of 100 mL min^-1^. A sample for UV–Vis analysis to measure the MB concentration was taken every 15 min. Fig. 14 shows the relative MB concentration over the time of the experiment and pictures of the MB solution at the beginning and the end of the experiment.

The experiment is divided into two parts. First, the UV reactor is off, and the adsorption of MB onto the surface of the fibers causes a drop in concentration. After 60 min the concentration starts to stabilize due to saturation effects. Then the UV reactor is switched on with an irradiation intensity of 30% (12.6 W), and photocatalytic degradation of MB starts. The concentration drops fast in the first 60 min after the UV reactor is switched on. Full MB degradation is observed after 180 min.

This result proves the reactor’s ability to be used for degradation experiments with micropollutants in water treatment. Furthermore, degradation experiments with other MPs (sulfamethoxazole, carbamazepine, diclofenac, and diatrizoate), other reactor geometries, hybrid processes with three-phase reactors, and different irradiation intensities were conducted. As these results exceed the scope of the manuscript, they are not presented here. They can be found in other publications or requested from the authors. The reactor was used for six months without any problems with an irradiation time of up to 6 h a day for water treatment experiments. Due to the elevated temperatures at the UV-LED-arrays, the lifetime of the LEDs was shorter than expected. After replacing the LEDs and adding the water cooling to the reactor, it is back in service for over three months before publication without any problems.

Overall, a lab-scale tubular UV reactor has been designed that is suitable to host different reactor geometries for different applications. Two of these devices have been built and used successfully for micropollutant degradation in water treatment over the last nine months within our labs. To further increase the efficiency of the reactor, reflectors can be added to the UV reactor next to the UV-LED-arrays, guiding a higher ratio of emitted UV light into the reactor to be used for the photocatalytic process [27]. Furthermore, these reflectors would protect the electronic components inside the UV reactor from UV radiation.

## CRediT authorship contribution statement

**Stefan Herrmann:** Conceptualization, Methodology, Investigation, Visualization, Writing – original draft, Writing – review & editing. **Lukas T. Hirschwald:** Methodology, Writing – review & editing. **Karl H. Heidmann:** Software, Validation. **John Linkhorst:** Conceptualization, Methodology, Writing – review & editing, Supervision. **Matthias Wessling:** Conceptualization, Writing – review & editing, Project administration, Funding acquisition.

## Declaration of competing interest

The authors declare that they have no known competing financial interests or personal relationships that could have appeared to influence the work reported in this paper.
